# Supplementary material for: Single-cell RNA sequencing analysis of shrimp immune cells identifies macrophage-like phagocytes
Source: eLife. 2022 Oct 6;11:e80127. doi: 10.7554/eLife.80127 (PMC9584607; doi:10.7554/eLife.80127)

Control: *Vibrio parahaemolyticus* (2×10^6 particles/g)

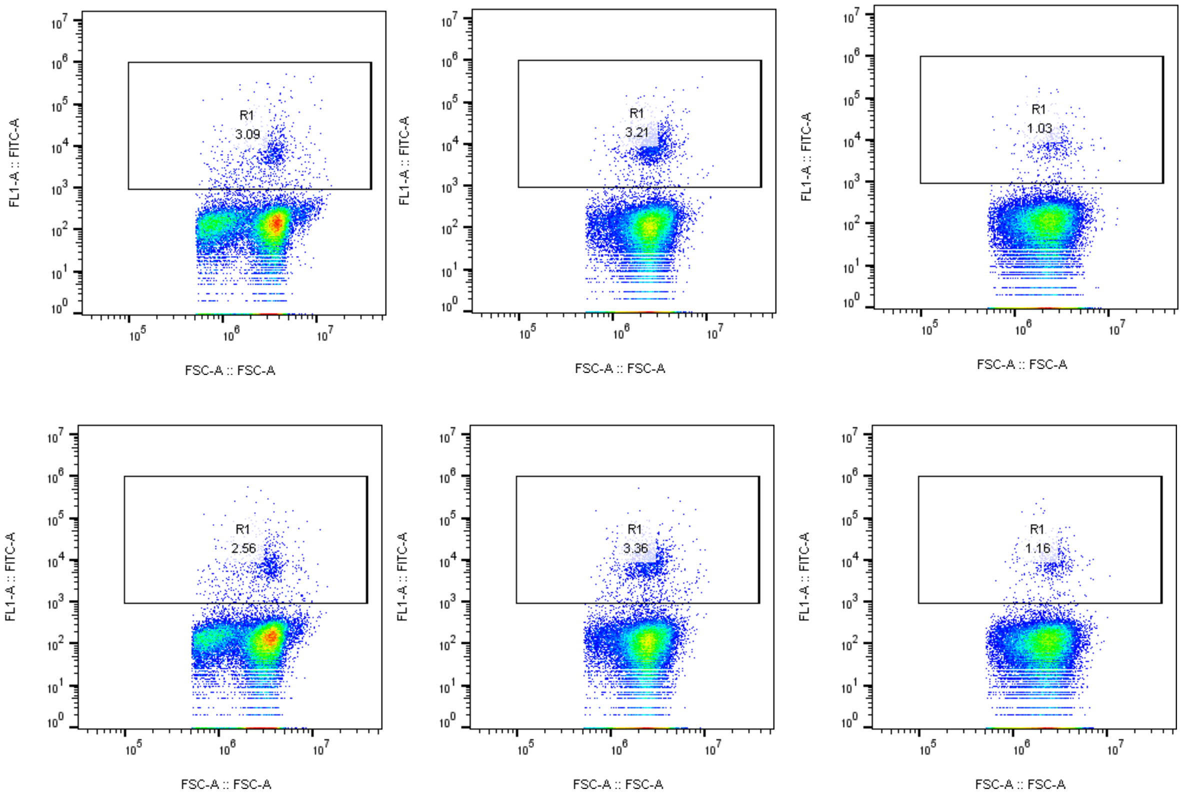

Cytochalasin D: *Vibrio parahaemolyticus* ($2×10^6$ particles/g) + cytochalasin D(5μM/g)

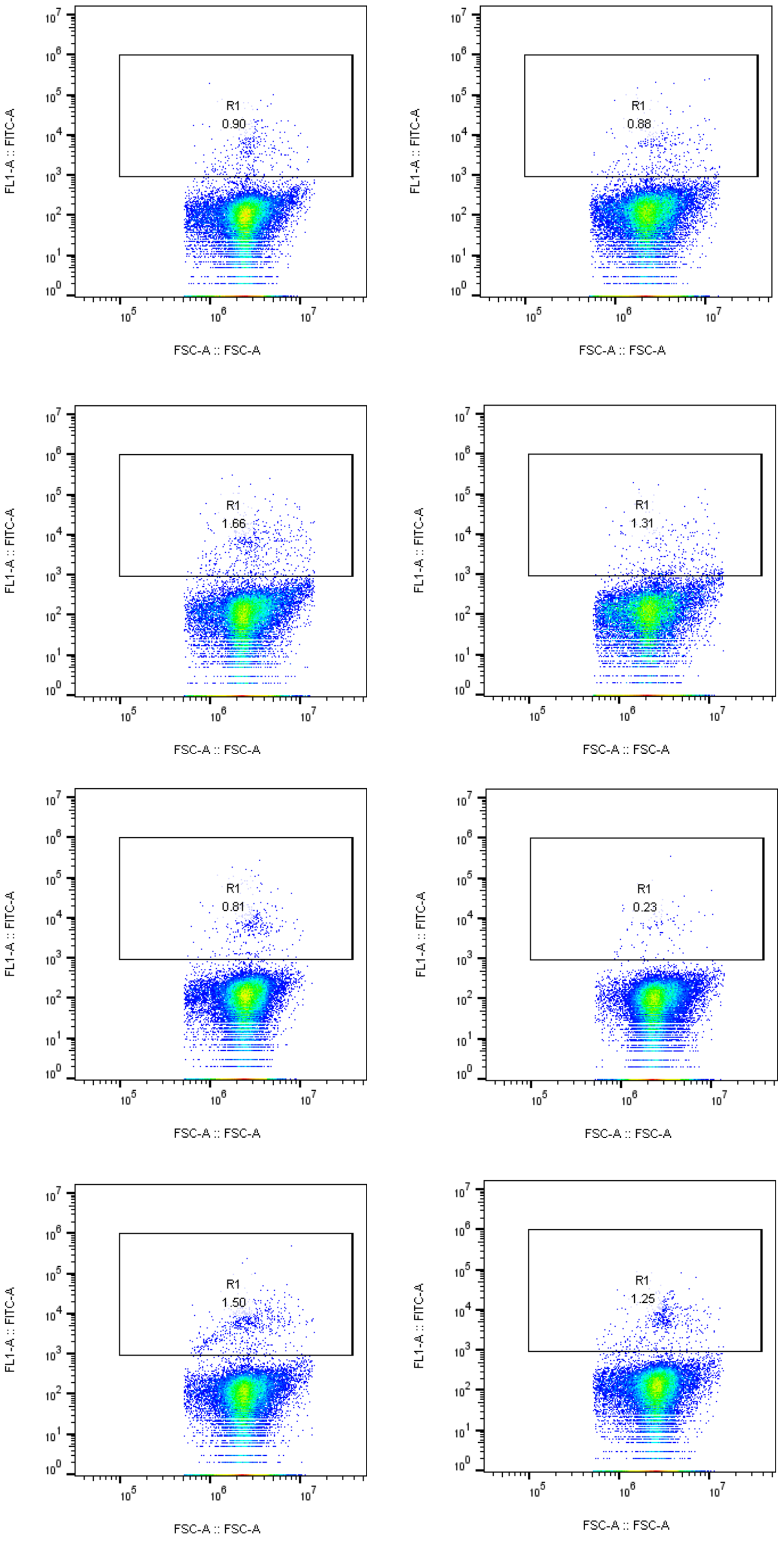

Supplement: Figure 3—source data 1. [file elife-80127-fig3-data1.pdf]
