# Supplementary figures and images for: Single-cell RNA sequencing analysis of shrimp immune cells identifies macrophage-like phagocytes
Source: eLife. 2022 Oct 6;11:e80127. doi: 10.7554/eLife.80127 (PMC9584607; doi:10.7554/eLife.80127)

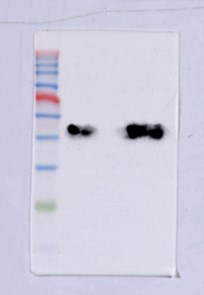

Supplement: Figure 3—source data 3. [file elife-80127-fig3-data3.zip › Source data/Figure 3-source data 10A.jpg]

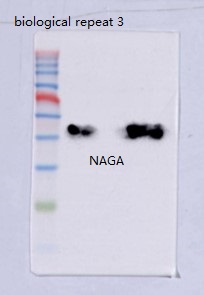

Supplement: Figure 3—source data 3. [file elife-80127-fig3-data3.zip › Source data/Figure 3-source data 10B.jpg]

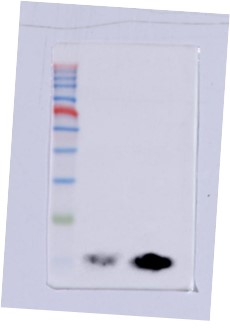

Supplement: Figure 3—source data 3. [file elife-80127-fig3-data3.zip › Source data/Figure 3-source data 11A.jpg]

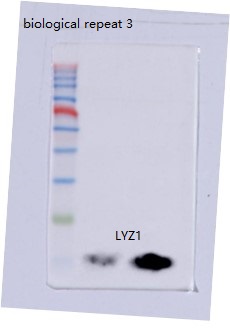

Supplement: Figure 3—source data 3. [file elife-80127-fig3-data3.zip › Source data/Figure 3-source data 11B.jpg]

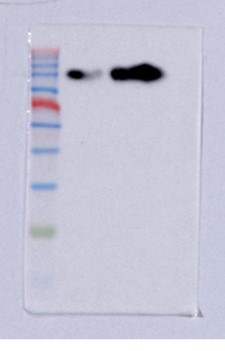

Supplement: Figure 3—source data 3. [file elife-80127-fig3-data3.zip › Source data/Figure 3-source data 12A.jpg]

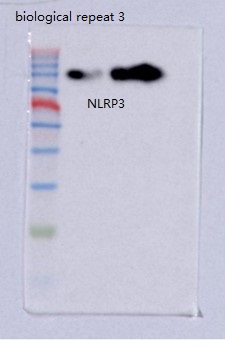

Supplement: Figure 3—source data 3. [file elife-80127-fig3-data3.zip › Source data/Figure 3-source data 12B.jpg]

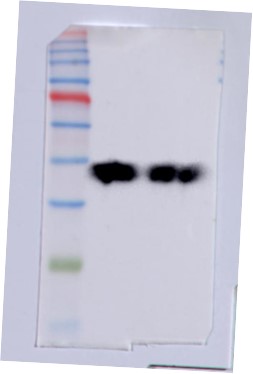

Supplement: Figure 3—source data 3. [file elife-80127-fig3-data3.zip › Source data/Figure 3-source data 1A.jpg]

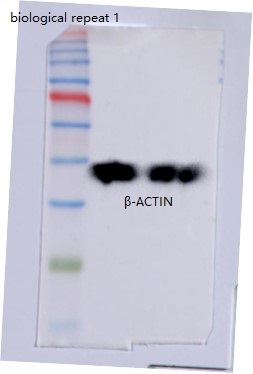

Supplement: Figure 3—source data 3. [file elife-80127-fig3-data3.zip › Source data/Figure 3-source data 1B.jpg]

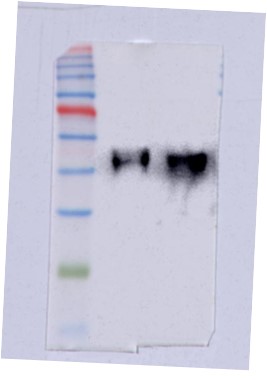

Supplement: Figure 3—source data 3. [file elife-80127-fig3-data3.zip › Source data/Figure 3-source data 2A.jpg]

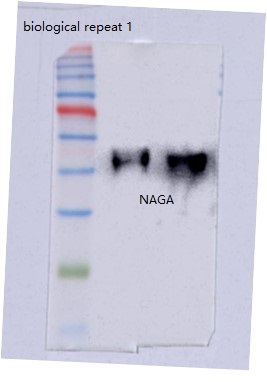

Supplement: Figure 3—source data 3. [file elife-80127-fig3-data3.zip › Source data/Figure 3-source data 2B.jpg]

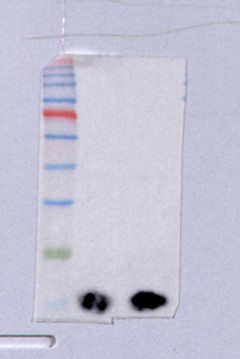

Supplement: Figure 3—source data 3. [file elife-80127-fig3-data3.zip › Source data/Figure 3-source data 3A.jpg]

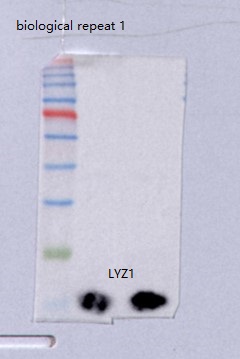

Supplement: Figure 3—source data 3. [file elife-80127-fig3-data3.zip › Source data/Figure 3-source data 3B.jpg]

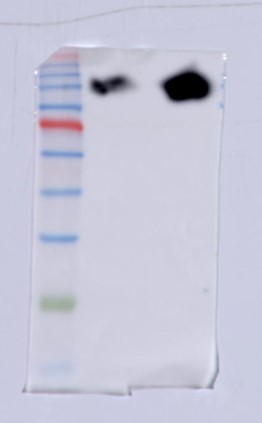

Supplement: Figure 3—source data 3. [file elife-80127-fig3-data3.zip › Source data/Figure 3-source data 4A.jpg]

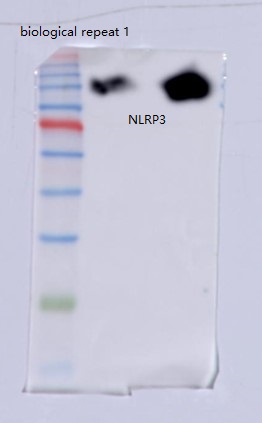

Supplement: Figure 3—source data 3. [file elife-80127-fig3-data3.zip › Source data/Figure 3-source data 4B.jpg]

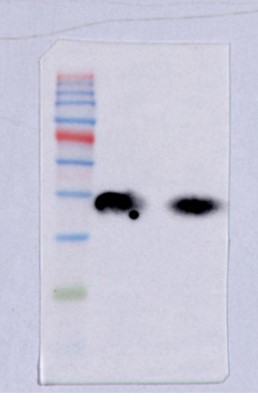

Supplement: Figure 3—source data 3. [file elife-80127-fig3-data3.zip › Source data/Figure 3-source data 5A.jpg]

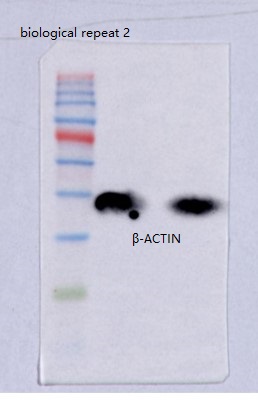

Supplement: Figure 3—source data 3. [file elife-80127-fig3-data3.zip › Source data/Figure 3-source data 5B.jpg]

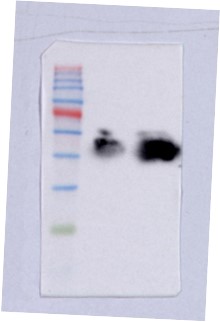

Supplement: Figure 3—source data 3. [file elife-80127-fig3-data3.zip › Source data/Figure 3-source data 6A.jpg]

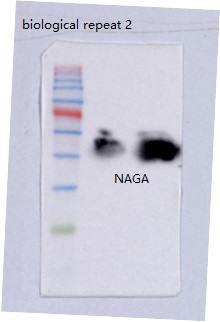

Supplement: Figure 3—source data 3. [file elife-80127-fig3-data3.zip › Source data/Figure 3-source data 6B.jpg]

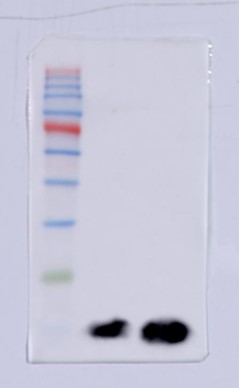

Supplement: Figure 3—source data 3. [file elife-80127-fig3-data3.zip › Source data/Figure 3-source data 7A.jpg]

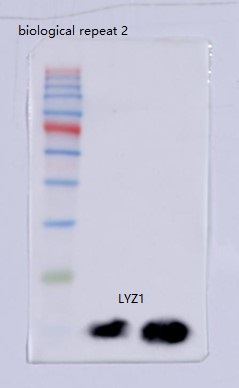

Supplement: Figure 3—source data 3. [file elife-80127-fig3-data3.zip › Source data/Figure 3-source data 7B.jpg]

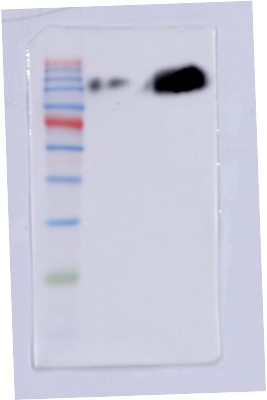

Supplement: Figure 3—source data 3. [file elife-80127-fig3-data3.zip › Source data/Figure 3-source data 8A.jpg]

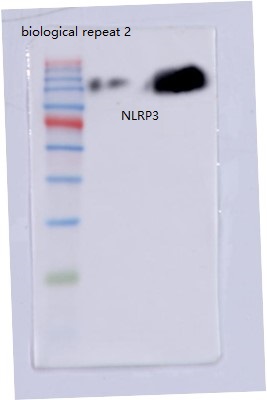

Supplement: Figure 3—source data 3. [file elife-80127-fig3-data3.zip › Source data/Figure 3-source data 8B.jpg]

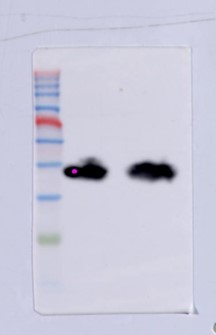

Supplement: Figure 3—source data 3. [file elife-80127-fig3-data3.zip › Source data/Figure 3-source data 9A.jpg]

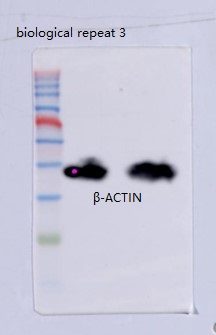

Supplement: Figure 3—source data 3. [file elife-80127-fig3-data3.zip › Source data/Figure 3-source data 9B.jpg]
